# INCREASING THE POWER OF INTERGENERATIONAL NETWORKS: ADVANCING A NEW EVALUATION TOOL

**DOI:** 10.1093/geroni/igz038.2485

**Published:** 2019-11-08

**Authors:** Shannon Jarrott, Lisa A Juckett, Jill Juris Naar

**Affiliations:** 1 The Ohio State University, Columbus, Ohio, United States; 2 Appalachian State University, Boone, North Carolina, United States

## Abstract

According to a 2018 national survey of intergenerational (IG) care providers, practitioners identified as their number one concern a need for evidence-informed evaluation tools to demonstrate their impact on older and younger participants. The Best Practices Checklist is a 14-item (yes/no) measure grounded in evidence of effective intergenerational strategies. Trained evaluators complete the checklist based on their observations of facilitators’ behaviors during IG activities. Exploratory factor analysis (promax rotation) of the Checklist for 132 IG activities offers insight to factor structure and item construction. An adequate two-factor structure was achieved; seven Checklist items were retained with factor loadings greater than .39. Seven items were deleted due to non-variance, high missing data, or double loading across factors. Factors reflect dimensions of: (a) person-centered strategies (e.g. selecting activities based on participants’ interests) and (b) creating a positive physical environment (e.g., grouping participants into intergenerational pairs or small groups). Findings indicate that a Best Practices Checklist with fewer items may offer a suitable tool for assessing the utilization of Best Practices during IG activities. Given the demand for IG evaluation tools, the 7-item BP Checklist can be a brief, easy-to-use measure that documents IG facilitators’ implementation of evidence-informed practices. Its use could be especially helpful if connected to varied indicators of program effectiveness and participant outcomes.

